# Supplementary material for: Direct measurement of nanostructural change during in situ deformation of a bulk metallic glass
Source: Nat Commun. 2019 Jun 4;10:2445. doi: 10.1038/s41467-019-10416-5 (PMC6547718; doi:10.1038/s41467-019-10416-5)
Supplement: Supplementary file 1 — Supplementary Information [file 41467_2019_10416_MOESM1_ESM.pdf]

Direct measurement of nanostructural change during *in situ* deformation of a bulk metallic glass - Supplementary Information

Pekin, *et al.*

Supplementary Figures

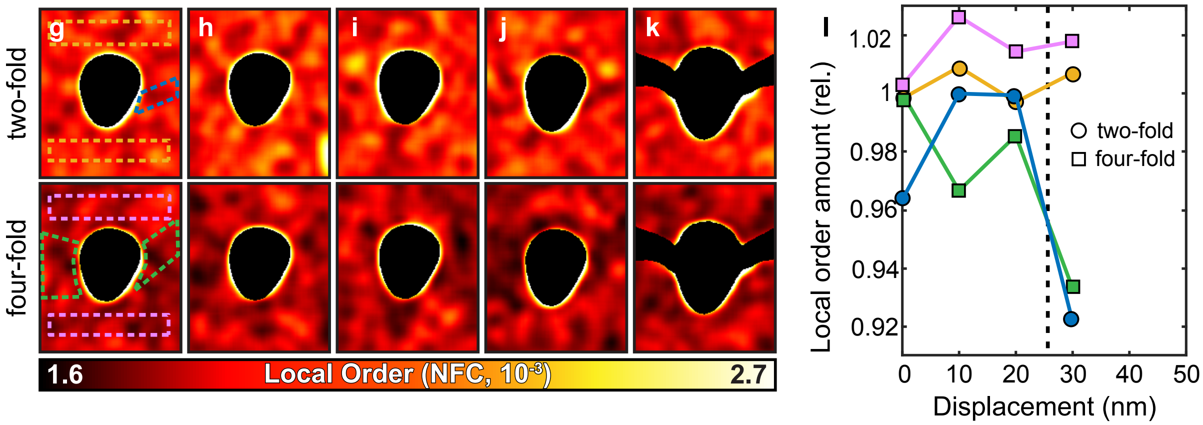

Supplementary Figure 1: A similar measurement of reduction of local order as Fig. 2, but using different areas. The two-fold polygonal area (blue) comprises 649 diffraction patterns, and the four-fold polygonal areas (green) comprise 3,858 patterns, while the combined rectangular areas (yellow and pink) comprise 5,934 patterns. The larger area covered and lower stress state explains the smaller fluctuations in local order amount.

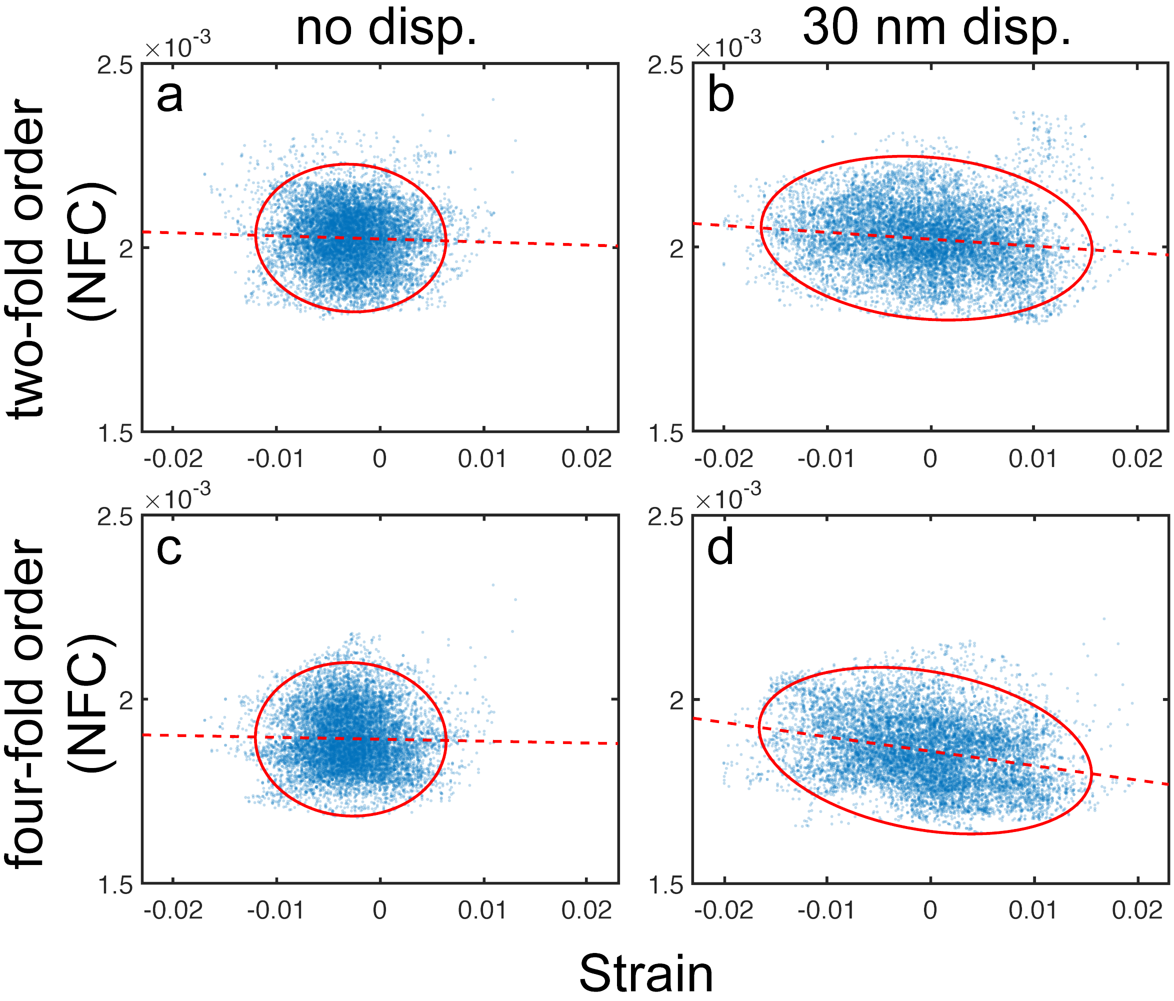

Supplementary Figure 2: a) Two and c) four-fold normalized Fourier coefficients plotted versus strain over the whole field of view, at zero deformation (corresponding to Fig. 2a). b) Two and d) four-fold normalized Fourier coefficients at 30 nm displacement (corresponding to Fig. 2d). The red ellipses are the 95% confidence ellipses, and the dashed line is the linear least squares fit to the data. The angle of the major ellipse axes, and dashed line in both b) and d) under deformation illustrate a negative correlation between strain and order.

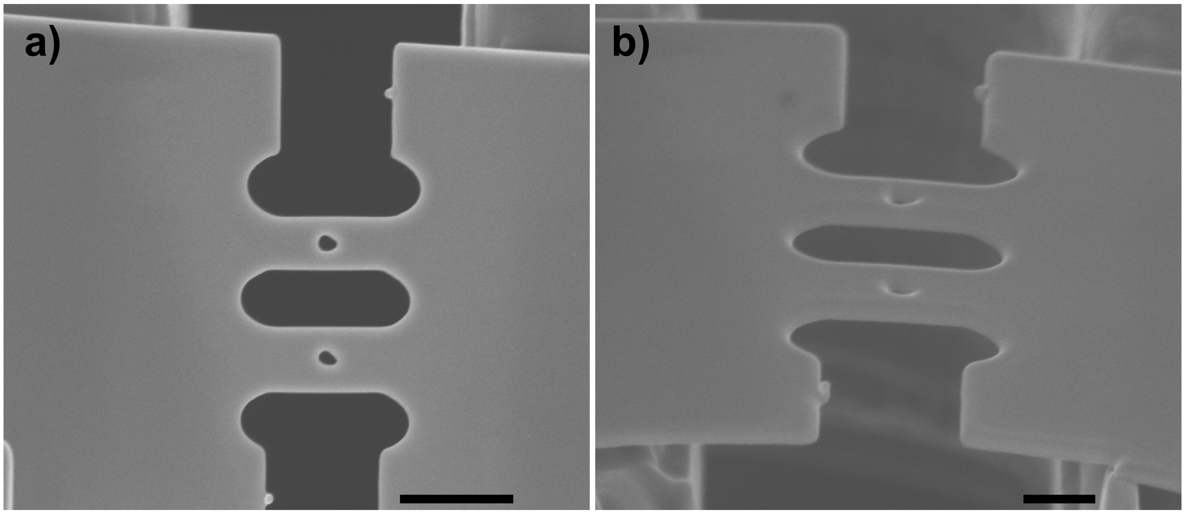

Supplementary Figure 3: The sample after focused ion beam milling on the Hysitron push to pull chip. **a**. Top down view. The scale bar is 1 micron. **b**. View at 52 degrees of stage tilt. The scale bar is 500 nm. The nominal thickness is approximately 80-90 nm. Holes were milled in the center of the tensile specimen in order to concentrate stress and strain.

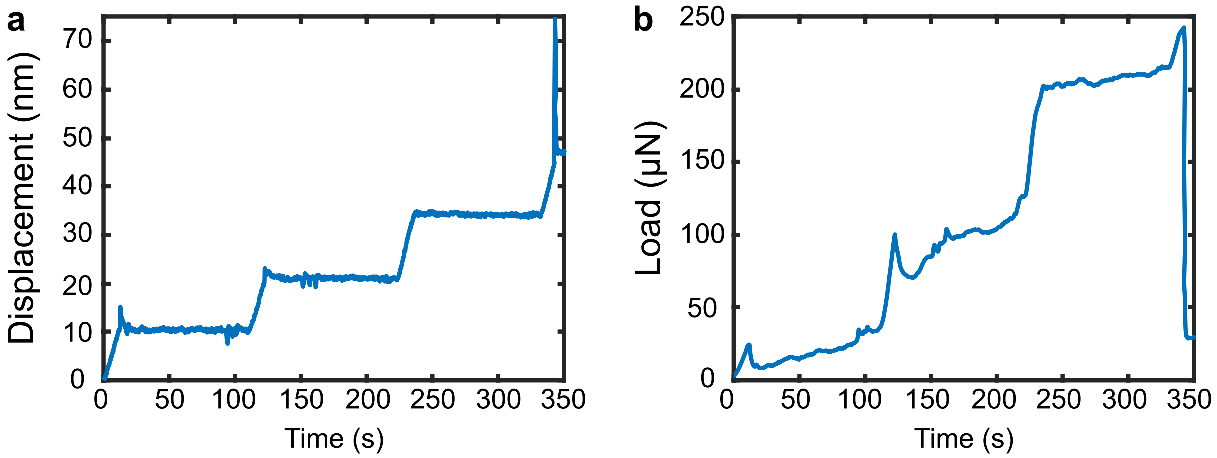

Supplementary Figure 4: a. Raw displacement *vs.* time plot until after fracture at roughly 340 seconds. The test was run under displacement control. The flat regions correspond to where scans b, c, and d were acquired. **b.** Raw load *vs.* time plot for the same time period. The sections corresponding to the flat segments in the displacement plot clearly show some linear load drift with respect to time.

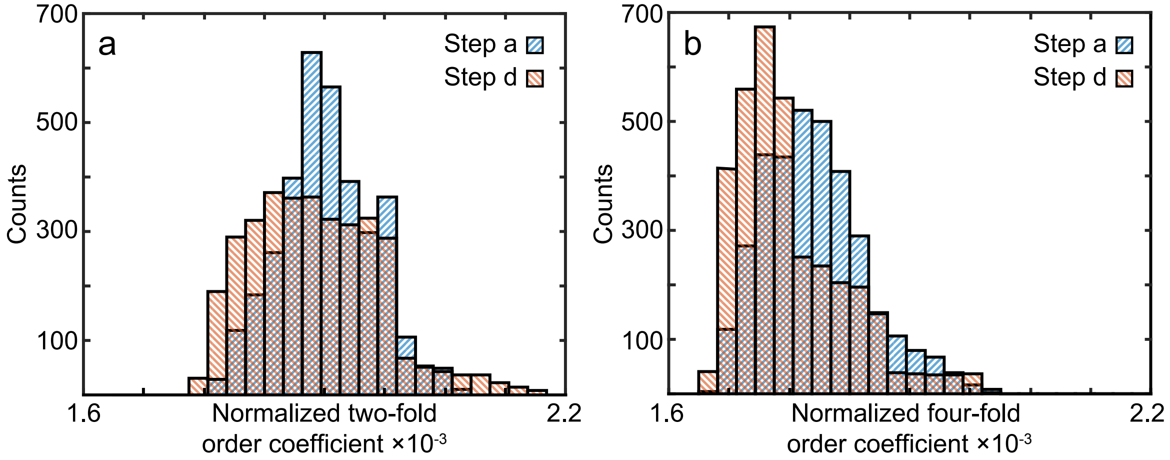

Supplementary Figure 5: Histograms showing the reduction in order in the a) two-fold and b) four-fold order maps in Fig. 2, corresponding to deformations a and d. The order coefficient values follow the normal distribution, and the two-sample *t*-test rejected the null hypothesis that the data came from distributions with equal means and unequal variances, with *p*-values of less than 0.00001.

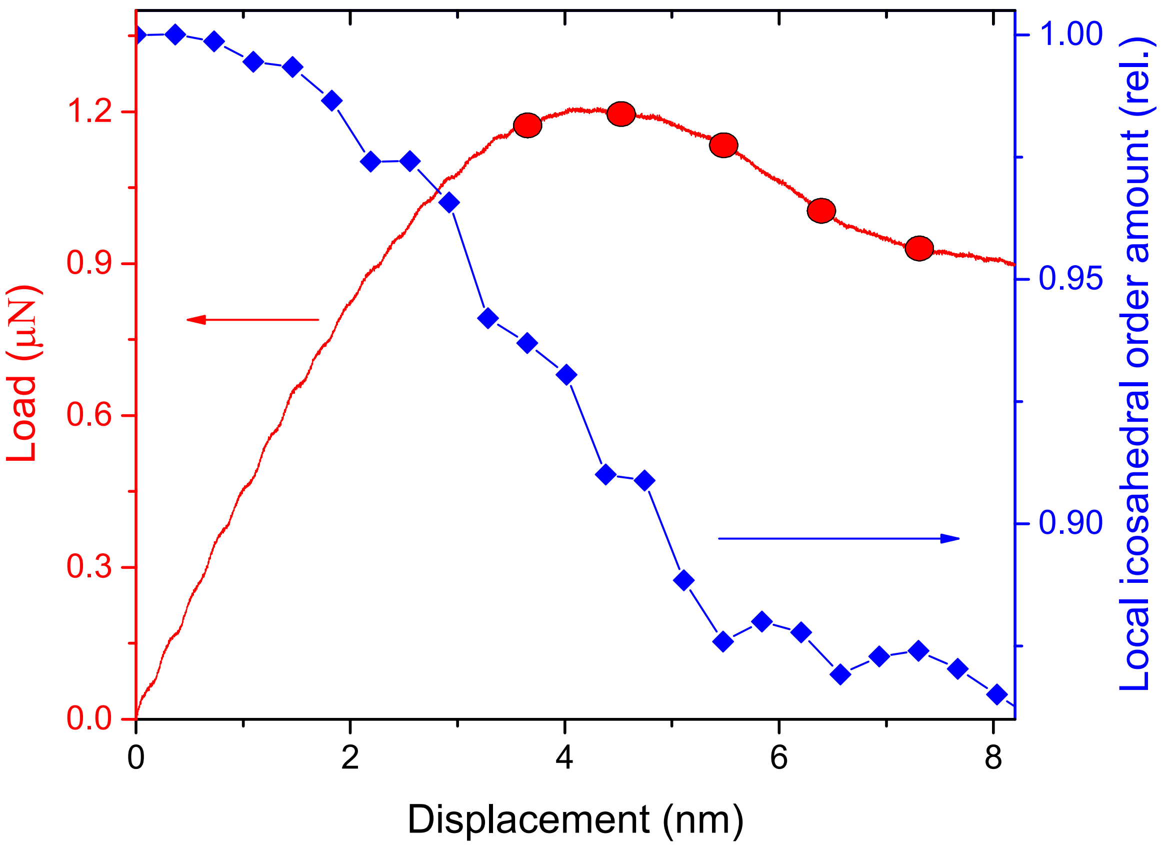

Supplementary Figure 6: Load-displacement curve (red) and corresponding fraction of local icosahedral order (blue) for the molecular dynamics simulation. The red circles correspond to a-e in Fig. 3.

Supplementary Note 1

In order to statistically measure the reduction in order in the two- and four-fold order maps in Fig 2l, basic statistical methods were performed.

A standard two-sample t-test was performed in MATLAB, which tests the null hypothesis that the data in vectors x and y come from independent random samples from normal distributions with equal means and unequal variances, using the first and fourth step of deformation (just prior to fracture) as x and y. In both two-fold and four-fold symmetry orders, the null hypothesis was rejected in the masked areas with a p-value < 0.00001.

We have also plotted histograms of the two and four-fold order coefficients from the masked areas at the first and fourth step of deformation in Supplementary Fig. 5. The histograms were constructed using over 3,000 diffraction pattern coefficients. This graphically shows the same result as the t-test, which is that the means are clearly different, the distributions are normal, and the variances are unequal. It is easily observable that there is a noticeable reduction in order over the 3,000+ diffraction patterns.

Finally, we have plotted in Supplementary Fig. 1 a reproduction of the local order maps in Fig. 2, and a measurement taken from areas with the highest strains and highest decrease in local order. In these regions, we see a statistically relevant reduction of local order of ~7%. Additionally, we have plotted local order measurements from the lower stress rectangular areas, which show a lack of significant change in order when compared to the high strain regions. The local order amounts have been normalized by their respective maximum mean of the high-strain regions, such that the maximum local order in the high strain region is equal to 1.
